# The Effect of Mixed Doping on the Microstructure and Electrophysical Parameters of the Multicomponent PZT-Type Ceramics

**DOI:** 10.3390/ma13081996

**Published:** 2020-04-24

**Authors:** Dariusz Bochenek, Przemysław Niemiec, Grzegorz Dercz

**Affiliations:** Faculty of Science and Technology, Institute of Materials Engineering, University of Silesia in Katowice, 75 Pułku Piechoty 1a, 41–500 Chorzów, Poland; przemyslaw.niemiec@us.edu.pl (P.N.); grzegorz.dercz@us.edu.pl (G.D.)

**Keywords:** PZT-type materials, ferroelectrics, piezoelectrics, perovskite structure, doping

## Abstract

This work shows the influence of admixture on the basic properties of the multicomponent PbZr_1−x_Ti_x_O_3_ (PZT)-type ceramics. It presents the results of four compositions of PZT-type material with the general chemical formula, Pb_0.99_*M*_0.01_((Zr_0.49_Ti_0.51_)_0.95_Mn_0.021_Sb_0.016_W_0.013_)_0.9975_O_3_, where, in the *M* position, a donor admixture was introduced, i.e., samarium (Sm^3+^), gadolinium (Gd^3+^), dysprosium (Dy^3+^) or lanthanum (La^3+^). The compositions of the PZT-type ceramics were obtained through the classic ceramic method, as a result of the synthesis of simple oxides. The X-ray diffraction (XRD) pattern studies showed that the obtained multicomponent PZT materials have a tetragonal structure with a P4mm point group. The microstructure of the obtained compositions is characterized by a well crystallized grain, with clearly visible grain boundaries. The composition with the admixture of lanthanum has the highest uniformity of fine grain microstructure, which positively affects its final dielectric and piezoelectric properties. In the multicomponent PZT-type ceramic, materials utilize the mixed (acceptor and donor) doping of the main compound. This dopiong method has a positive effect on the set of the electrophysical parameters of ceramic materials. Donor dopants W^6+^ (at positions B) and *M*^3+^ = Sm^3+^, Gd^3+^, Dy^3+^, and La^3+^ (at positions A) increase the dielectric and piezoelectric properties, while the acceptor dopant Sb^3+^ (at positions B) increases the time and temperature stability of the electrophysical parameters. In addition, the suitable selection of the set of admixtures improved the sinterability of the ceramic samples, as well as resulted in obtaining the required material with good piezoelectric parameters for the poling process. This research confirms that all ceramic compositions have a set of parameters suitable for applications in micromechatronics, for example, as actuators, piezoelectric transducers, and precision microswitches.

## 1. Introduction

Among the ceramic materials with functional properties discovered, to date, ceramic materials, ceramic composites, and solid solutions obtained on the basis of PbZr_1−x_Ti_x_O_3_ (PZT), due to their optimal electrophysical properties, have been successfully and extensively used in modern microelectronics, invariably since the 1960s [1,2,3,4,5]. Depending on the percentage of Zr/Ti, PZT solid solutions have a perovskite-type structure with symmetry from a rhombic, rhombohedral, tetragonal system, or are distinguished by the coexistence of the tetragonal and rhombohedral phase (the so-called morphotropic region). The chemical composition with the above-mentioned crystallographic symmetry has different properties and is selected for specific applications. From the point of view of the versatility of applications, the most important are the PZT-type compositions from the morphotropic region [6,7,8,9].

There are many ways to optimize and improve the properties of ceramic materials, such as using modern technological methods, using a protective or special atmosphere during sintering at high temperatures, doping of the basic composition, creating multicomponent ceramic composites with different properties, etc. [10,11,12,13]. One of the effective methods for optimizing a number of electrophysical parameters of PZT-type ceramic materials (dielectric, piezoelectric, mechanical, electromechanical, etc.) is appropriate doping of the basic composition with many modifiers [14,15,16]. Admixtures introduced into the basic PZT composition are the source of spatial charge and the associated spatial charge field, which affect the electrophysical properties of ceramics. As a result of the doping of the PZT base composition, multicomponent systems have been obtained showing much better properties than the non-doped PZT materials [17,18]. In the case of compositions close to the morphotropic boundary, an increase in the number of components (dopant ions) causes the expansion of the morphotropic region [19,20,21,22,23,24].

As a result of donor doping (the degree of oxidation of dopant ions is greater than the replaced ions), there is an increase in the number of lead vacancies V_Pb_, which promotes the movement of ions in the crystal lattice under the influence of external interactions (the mobility of domain walls increases). The PZT-type solid solution doped in the above way shows a decrease in the field of spatial charge, acquiring ferroelectric soft properties [25].

As a result of acceptor doping (the degree of oxidation of dopant ions is smaller than the replaced ions), the ceramic material is characterized by increased ferroelectric hardness, which is associated with an increase in the number of V_O_ oxygen vacancies. The resulting oxygen vacancies, interacting with domain walls, reduce domain mobility, which results in increased structure stability (an increase in the so-called ferroelectric hardness of the material) [26]. As a result of ferro-hard doping, the field of spatial charge increases, fixing domains and hindering the movement of domain walls.

There are many works in which doped multicomponent PZT materials have been obtained, for example in [17,27,28,29,30]. In [31], the authors obtained a number of PZT-type compositions doped with single rare earths, using the sol-gel method. However, SEM studies have shown a high porosity of these materials with poorly crystallized and heterogeneous grains in volume. In turn, reference [32] presented another composition of PZT doped with single rare earths synthesized by the classic powder technology, however, with the presence of the pyrochlore phase and average values of piezoelectric parameters. Studies have shown that in order to obtain a number of profits in the final electrophysical parameters of PZT-type ceramic materials (for specific applications), multicomponent doping of the basic PZT composition can be effective.

For this purpose, the results are described for an investigation of the four compositions of multicomponent PZT ceramics with the general formula Pb_0.99_*M*_0.01_((Zr_0.49_Ti_0.51_)_0.95_Mn_0.021_Sb_0.016_W_0.013_)_0.9975_O_3_. The admixtures substituted in the B position of the compound (i.e., manganese Mn^4+^, antimony Sb^3+^, and tungsten W^6+^) are unchanged. Sb^3+^ is an acceptor admixture (increase of parameter stability), W^6+^ is a donor admixture (increase dielectric and piezoelectric properties), whereas the admixture of Mn^4+^ is intended to improve the ceramic microstructure (increasing grain uniformity in microstructure). The *M*^3+^ donor admixture (in the amount of 0.01) substituted in the A position of the compound was samarium Sm, gadolinium Gd, dysprosium Dy, or lanthanum La. The purpose of doping performed in this work was to examine the impact of the admixture mix used on the basic properties of the PZT-type ceramic materials. The set of admixtures introduced into the base composition was selected with the aimed to optimize the electrophysical properties and improve the sinterability of ceramics. Comprehensive analyzes and tests were carried out, for example, differential thermal, X-ray, surface morphology (SEM) with EDS tests, as well as dielectric, ferroelectric, piezoelectric properties, and direct current (DC) electrical conductivity.

## 2. Experiment

This work presents and compares the properties of four compositions of a multicomponent PZT material with a general chemical formula, i.e., Pb_0.99_*M*_0.01_((Zr_0.49_Ti_0.51_)_0.95_Mn_0.021_Sb_0.016_W_0.013_)_0.9975_O_3,_ where, in the *M* position, the following admixtures were introduced: samarium Sm^3+^, gadolinium Gd^3+^, dysprosium Dy^3+^, and lanthanum La^3+^, specified as P-Sm, P-Gd, P-Dy, and P-La, respectively.

The starting oxides were PbO (99.99% purity, POCH, Gliwice, Poland), ZrO_2_ (99.00% purity, Merck), TiO_2_ (99.99% purity, Merck, Darmstadt, Germany), MnO_2_ (99% purity, Aldrich, Saint Louis, MI, USA), Sb_2_O_3_ (99.995% purity, Aldrich, Saint Louis, MI, USA), and WO_3_ (99.9% purity, Fluka, Bucharest, Romania), as well as in the *M* position the following oxide series were introduced: Sm_2_O_3_ (99.5% purity, Aldrich, Saint Louis, MI, USA), Gd_2_O_3_ (99.5% purity, Aldrich, Saint Louis, MI, USA), Dy_2_O_3_ (99.9% purity, Aldrich, Saint Louis, MI, USA), and La_2_O_3_ (99.98% purity, Fluka, Shanghai, China). The PbO powder was weighed with allowance (5 wt %) to compensate for its losses during the technological process. The powders were mixed in a planetary mill Fritsch (Pulwerisette 6) for 24 h, in ethanol, using zirconium balls. Consecutively, the mixtures of powders were dried, and next, synthesized at the same conditions, 850 °C for 4 h using calcination. Sintering of the ceramic samples was carried out at 1150 °C for 2 h (heating rate of 150 °C/h), and next, samples were grinded and polished (to a thickness of 1 mm). After this, the ceramic samples were annealed to remove mechanical stress (at temperature 700 °C/15 min.). At the end of the technological process, the electrodes from the silver paste had been deposited onto both surfaces of the samples for electrical testing.

The synthesis temperature of the ceramic compositions was determined on the basis of the differential thermal analysis (DTA), thermogravimetric (TG), and differential thermogravimetric (DTG) analysis. DTA, DTG, and TG tests were carried out using the Q-1500D derivatograph (Paulik-Paulik-Erdey system derivative (MOM, Q-1500D, F. Paulik, J. Paulik, L. Erdey system, Budapest, Hungary) in the temperature range from 20 to 1050 °C. 

The X-ray diffraction (XRD) patterns of the PZT-type ceramic powders were measured at room temperature using a PANalytical, Phillips X’Pert Pro, Eindhoven, The Netherlands (λCuK*_α_*_1_ = 1.54056 Ǻ). All X-ray diffraction patterns were registered in 2θ range 10 to 135°, in step-scan mode 0.05° and 4 s/step. The surface morphology (microstructure) series of the samples and chemical composition were measured by scanning electron microscopy JSM-7100F TTL LV (Tokyo, Japan) with the EDS analysis system (Tokyo, Japan). The temperature measurements of dielectric properties were performed using a QuadTech 1920 Precision LCR meter, Quad/Tech, Inc., Maynard, MA, USA (temperature range from 20 to 450 °C). The temperature tests of DC electric conductivity were measured using a 6517B Keithley electrometer (Cleveland, OH, USA) (temperature range from 20 to 450 °C). The ferroelectric measurements (hysteresis loops) were investigated with a Sawyer–Tower circuit and a Matsusada Inc. HEOPS-5B6 Precision high voltage amplifier (Kusatsu, Japan). The data were stored on a computer disc using an A/D, D/A transducer card (National Instruments Corporation, Austin, TX, USA) and the LabView computer program.

The poling process of the ceramic samples was carried out on a Matsusada Precision Inc. HEOPS-5B6 high voltage supply (Kusatsu, Japan), by the high voltage method at silicon oil and with the following poling conditions: electric field *E_pol_* = 2.5 kV/mm, temperature *T_pol_* = 120 °C, and time *t_pol_* = 0.5 h. The piezoelectric parameters were calculated using the resonance–antiresonance method, while the piezoelectric coefficient *d*_33_ was measured using a YE2730A d33 meter (APC International Ltd., Mackeyville, (PA), USA) at room temperature.

## 3. Results and Discussion

**Figure 1:**
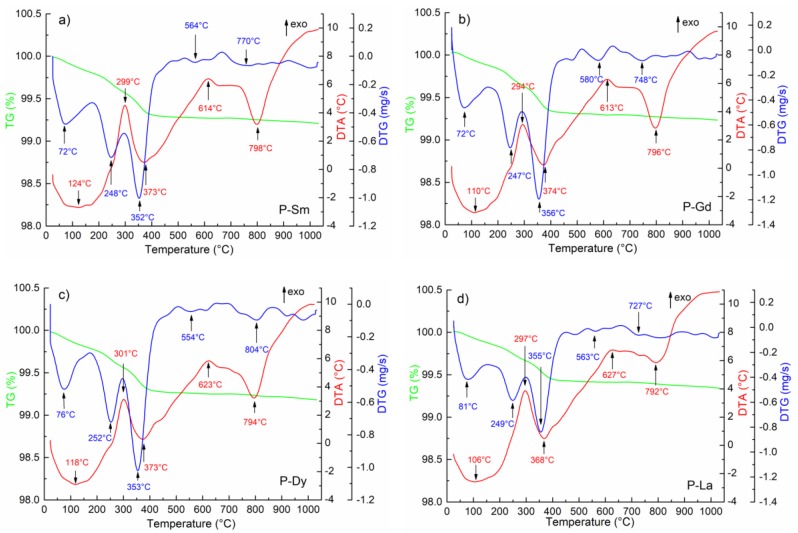
Differential thermal analysis (DTA), thermogravimetric (TG), and differential thermogravimetric (DTG) analysis of the PbZr_1−x_Ti_x_O_3_ (PZT)-type powders, (**a**) P-Sm, (**b**) P-Gd, (**c**) P-Dy, (**d**) P-La.

The DTA and TG tests performed for all PZT-type compositions show similar characteristic maxima and minima on their curves (Figure 1). On the TG curves, for all analyzed powder samples, the largest weight loss is observed in the temperature range from room temperature to approximately 400 °C (for P-Sm up to 391 °C, for P-Gd up to 394 °C, for P-Dy to 397 °C, and for P-La to 395 °C). These temperature ranges correspond to the local minima on the DTG curve, in which the weight loss occurs faster. The first weight loss (below 150 °C) is attributed to the vaporization of water, while the second one (up to approximately 400 °C) is related to the elimination of organic residual from the milling process [33]. The ceramic samples show very low total weight loss Δ*m*, i.e., 1.0%, 0.8%, 0.8%, and 0.7% for P-Sm, P-Gd, P-Dy, and P-La samples, respectively. Such a small weight loss and the DTA curves indicate that the synthesis process was completely correct.

On the DTA curves, three endothermic minima and two exothermic maxima can be observed, corresponding to the thermal processes occurring in the stoichiometric mixture of the PZT-type compositions. The first endothermic peaks which occur above 100 °C correspond to the evaporation of residual moisture from the powder samples. The successive peaks correspond to the creation of intermediate phases. The exothermic peak at the temperature range from 294 to 301 °C is related to the phase transition of lead oxide with increasing temperature [34]. The peak occurring above 614 °C (for P-Sm), 613 °C (for P-Gd), 623 °C (for P-Dy), and 627 °C for P-La) initiates gradually occurring reactions in the material (e.g., PT). Next, as a result of the reaction, the main perovskite phase is formed. In the case of the PZT-type ceramic materials, the temperature area formation of the perovskite phases is below 800 °C (endothermic peak at 798 °C for P-Sm, 796 °C for P-Gd, 794 °C for P-Dy, and 792 °C for P-La). Above this temperature (indicating the end of phase formation), no further changes occur. On the basis of the DTA and TG tests for all ceramic compositions, the synthesis conditions selected were 850 °C/4 h. 

**Figure 2:**
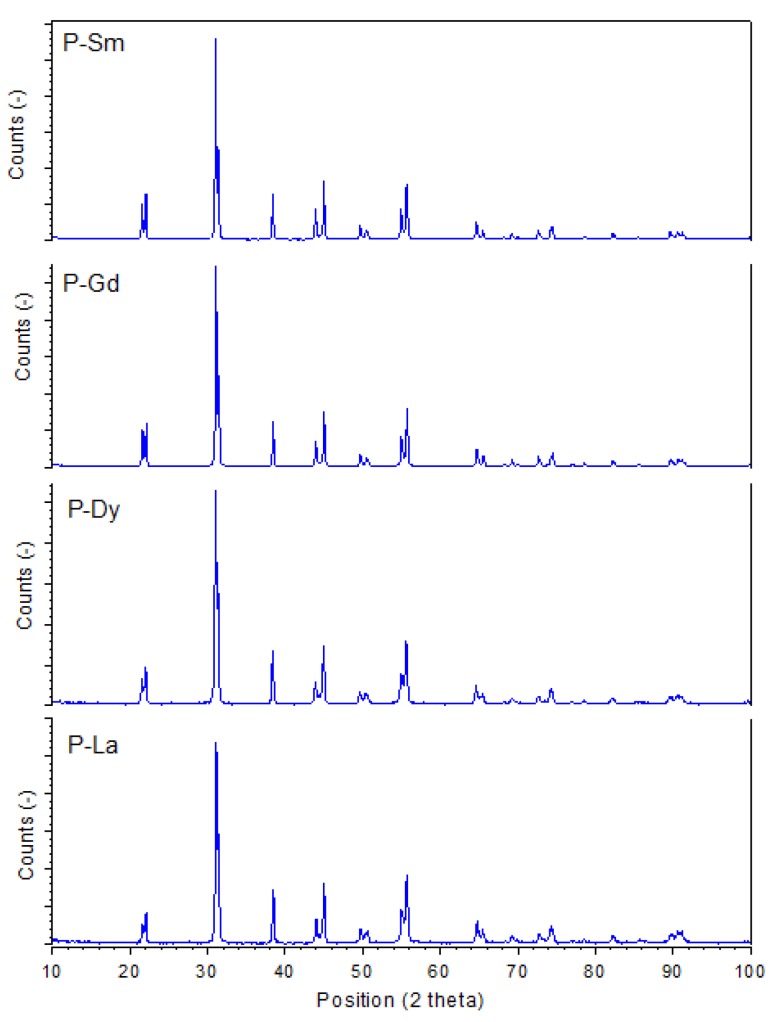
X-ray diffraction (XRD) pattern measurements of the multicomponent PZT-type materials.

Figure 2 shows the X-ray diffraction patterns of the PZT-type materials. All diffraction lines, measured at room temperature, have been identified as peaks belonging to the tetragonal perovskite structure. The XRD studies also confirmed that the materials have a single phase, without the presence of other (additional) phases (e.g., the pyrochlore phase). The Rietveld method [35] was used to calculate the unit cells for the analyzed samples. For all samples, the indices of crystallographic plane for diffraction peaks are indexed with space group P4mm. A change of the type of admixture in the main Pb(Zr_0.49_Ti_0.51_)O_3_ composition causes a change in the values of the parameters of elementary cell (Table 1). The largest volume of the unit cell is shown by the P-Dy sample (*V* = 67.202 Å^3^), with the unit cell parameters *a*_0_ and *c*_0_ of 4.0363 Å and 4.1249 Å, respectively. In turn, the smallest volume of a unit cell is shown by the P-La sample (*V* = 66.884 Å^3^), with the unit cell parameters *a*_0_ and *c*_0_ of 4.0314 Å and 4.1154 Å, respectively (lower height of the cuboid unit cell). Spontaneous deformation of the tetragonal unit cell *δ*_T_ calculated from Equation (1) is 0.015042 for P-Sm, 0.015159 for P-Dy, 0.014580 for P-Gd, and 0.013843 for P-La.

(1) $$\delta_{T}=\frac{c_{0}-{\bar{a}}_{0}}{{\bar{a}}_{0}}$$

were ${\bar{a}}_{0}=\sqrt[{3}]{V}$, *c*_0_ is the unit cell parameter, and *V* is the unit cell volume.

In the case of the analyzed samples, the calculated tetragonality (c/a ratio), shown in Table 1, maintains a certain tendency (except the P-Dy sample), i.e., slightly decreases with an increase of the ionic radius (*r*_Sm_ = 1.24 Å, *r*_Gd_ = 1.107 Å, *r*_Dy_ =1.083 Å, and *r*_La_ = 1.36 Å). Considering the ionic radii of the *M* dopants added to the main chemical compound (Sm, Gd, Dy, and La), they substitute in the A position (in place of Pb, *r*_Pb_ = 1.49 Å). In the case of the Mn (*r*_Mn_ = 0.53 Å), Sb (*r*_Sb_ = 0.76 Å), and W (*r_W_* = 0.60 Å) admixtures substitute in the B (Zr/Ti) positions (*r_Zr_* = 0.72 Å and *r_Ti_* = 0.605 Å) of the main chemical compound.

The admixtures substituting in both the A and B positions of the compound with different ionic radii cause local crystal lattice distortions and generate leads/oxygens vacancies.

The following additional structural parameters were calculated on the basis of X-ray tests and Equations (2)–(4), i.e., minimal possible size of crystals (Equation (2)), the dislocation density (Equation (3)) and the microstrain (Equation (4)) of the PZT-type ceramic samples. A minimal possible size of the crystals of the ceramic materials was calculated on the basis of the full width at half maximum (FWHM) originating from the main perovskite X-ray diffraction peak (110), according to the Scherrer Equation (2):

(2) $$D=\frac{K\lambda }{\beta \cos\theta }$$

where *D* is the diameter of the particles, *K* is the dimensionless shape factor (usually its value is 0.9), *λ* is the X-ray wavelength, *β* is the size contribution to the full width at half maximum of the main diffraction peak (110), and *θ* is the Bragg angle [36]. The Scherrer method is based on the relationship between the size of crystallites and the broadening of the profile of diffraction lines. The greater the reflex extension, the smaller the grain sizes in the material. The calculated values of the crystallite sizes, *D*, are 44 nm for the P-Sm sample, 41 nm for the P-Gd sample, 27 nm for the P-Dy sample, and 29 nm for the P-La sample. 

The amounts of defects in the crystal is estimated as the length of dislocation lines per unit volume. The dislocation density *δ* was calculated according to Equation (3) [37]:

(3) $$\delta =\frac{1}{D^{2}}$$

where as the structural parameter, microstrain *ξ,* was calculated from Equation (4) [38]:

(4) $$\xi =\frac{\beta }{4\tan\theta }$$

The calculated set of the structural parameters of the obtained ceramic samples are summarized in Table 1.

**Figure 3:**
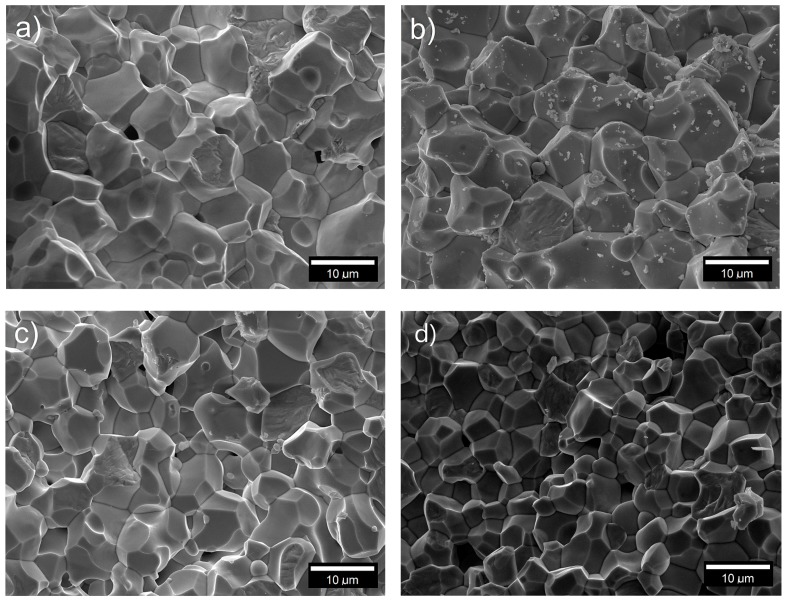
SEM images of the microstructure of the multicomponent PZT-type samples fractures. (**a**) samarium Sm^3+^ (P-Sm); (**b**) gadolinium Gd^3+^ (P-Gd); (**c**) dysprosium Dy^3+^ (P-Dy); (**d**) lanthanum La^3+^ (P-La).

The microstructural SEM images of the obtained PZT ceramic compositions (Figure 3) are characterized by a densely packed grain, with fine crystallized grains, but with a significant heterogeneity of the shape and size thereof. The boundaries of the layers between the grains are perfectly visible. During the fracture of the samples, the grain-bound cracking mechanism (inter-grain cracking mechanism) dominates, however, there is also (to a small extent) a grain-breaking mechanism (trans-grain cracking mechanism) [39,40]. This indicates a very high mechanical strength of the interior of the ceramic grains of the samples. A durable material inside the grains indicates the correctness of the process for obtaining the ceramic material, with correctly selected technological conditions. However, in the case of the PZT-Gd material, small debris from fragments of cracked grains is observed on the microstructural SEM image of the fracture of the sample. Such a phenomenon also requires the optimization of technological conditions in order to increase the strength of microstructure grains (e.g., by slightly modifying the temperature or sintering time). The microstructure of PZT-La ceramics has the finest grain ($\bar{r}$ ~ 7.6 μm), which also has the largest degree of grain size uniformity. The largest grains ($\bar{r}$ ~ 9.2 μm) have the microstructure of PZT-Sm ceramics (Table 1).

**Figure 4:**
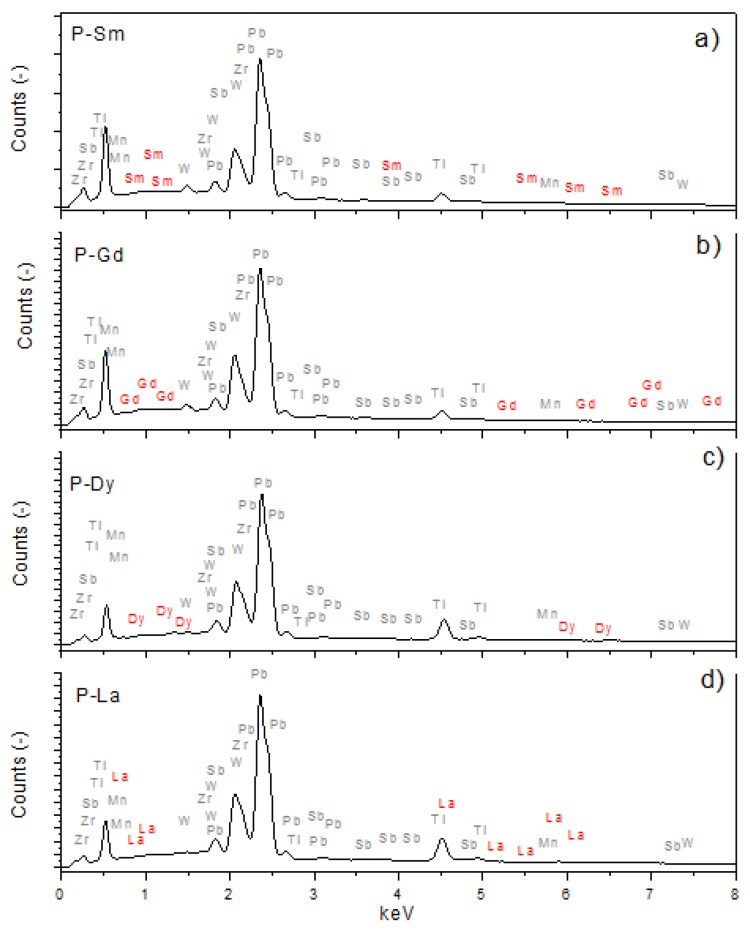
The EDS analysis of the element distribution for the multicomponent PZT-type ceramics. (**a**) P-Sm, (**b**) P-Gd, (**c**) P-Dy, (**d**) P-La.

The EDS analysis (Figure 4, Table 2) was performed on five randomly selected measurement microareas of the surface samples. The EDS analysis showed that the ceramic samples were free of impurities and that the chemical composition was close to the assumed one. For all analyzed compositions, there is a slight excess of lead (Pb) and a slight deficiency of zirconium (Zr). In the case of the remaining elements, no identical trend is observed for all analyzed compositions. In the case of the P-Sm sample, an excess for tungsten (W) and samarium (Sm) was revealed, while a deficiency for titanium (Ti), manganese (Mn), and antimony (Sb) was noted. In the case of the P-Gd sample, an excess for tungsten (W), titanium (Ti), and gadolinium (Gd) was revealed, while a deficiency for manganese and antimony (Sb) was noted. Excess for tungsten (W) and dysprosium (Dy) was revealed for the P-Dy sample, while the deficiency for titanium (Ti), manganese (Mn), and antimony (Sb) was noted. In the case of the P-La sample, a deficiency for tungsten (W), antimony (Sb), titanium (Ti), and manganese (Mn) were revealed, while the amount of lanthanum (La) was equal. All deviations from the assumed theoretical compositions are within error limits.

**Figure 5:**
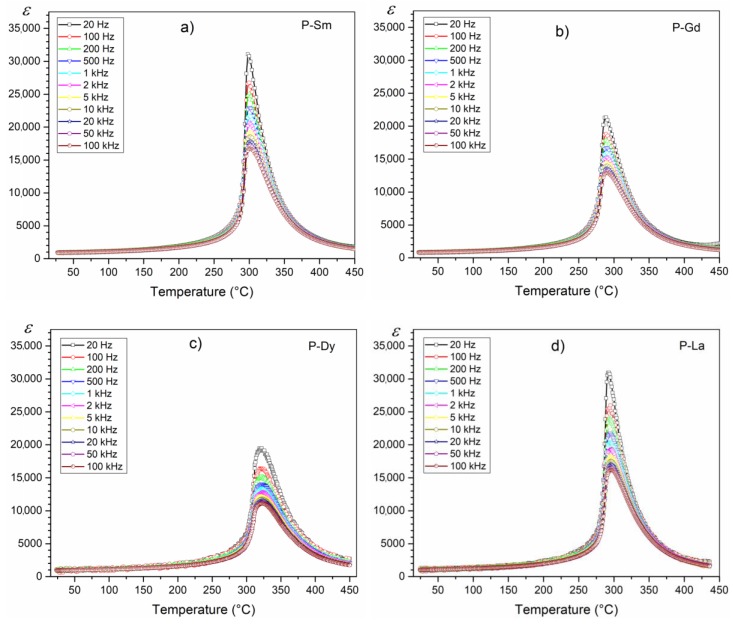
Temperature dependencies of the electric permittivity of the following multicomponent PZT-type samples: (**a**) P-Sm, (**b**) P-Gd, (**c**) P-Dy, (**d**) P-La.

Figure 5 presents temperature diagrams of electric permittivity for the tested doped PZT compositions. In the case of the P-Sm and P-La compositions, a fairly sharp phase transition with high values of maximum electric permittivity is observed. The increase in the frequency of the measuring field significantly reduces the value of the maximum electric permittivity, and at the same time does not cause a shift in the phase transition temperature with an increase in the frequency of the measuring field (no frequency dispersion characteristic of relaxation materials) [41,42].

In the case of the P-Dy composition on the temperature graphs *ε*(*T*), the dielectric peaks are broadened which could be due to the characteristic behavior of disordered perovskite structure with diffuse phase transition (from the examined group of samples the diffuse phase transition is the largest). In addition to the diffuse phase transition and the lowering of the electric permittivity value, the P-Dy composition has a lower frequency susceptibility to the electric permittivity value (reduction of electric permittivity, with the frequency increase is not as high as in the case of the P-Sm and P-La compositions).

The degree and nature of the diffuse phase transition is not the same for all multicomponent ceramic materials with the perovskite structure [43], and its course is influenced by various technological process factors, for example, fluctuations in the chemical composition [44] or substitution disordering in the arrangement of cation in one or more crystallographic sites [20] in the structure, leading to a microscopic heterogeneity in the composition (microareas as the centers of new phase formation), and thus in a distribution of different local Curie points [43] or the heterogeneity of defect distribution and mechanical stress in the ceramic grains. 

**Figure 6:**
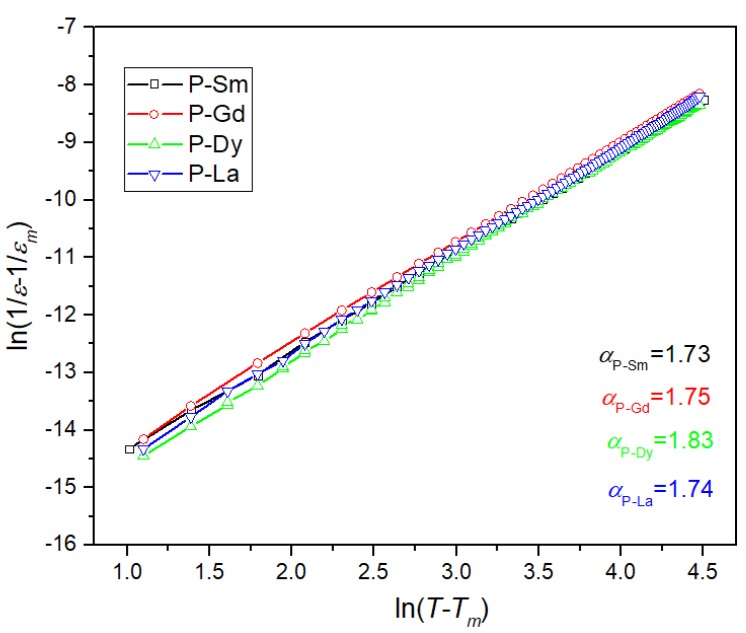
Plots of the ln(1/*ε* – 1/*ε*_m_) vs. ln(*T* – *T*_m_) for the PZT-type samples (paraelectric phase).

In perovskite materials, the following relationship is used to determine the degree of diffuse phase transition [45]:

(5) $$\frac{1}{\varepsilon }-\frac{1}{\varepsilon_{m}}=C{\left(T-T_{m}\right)}^{\propto }$$

where *C* is the temperature constant, *ε*_m_ is the maximum electric permittivity (at the temperature *T*_m_), *T*_m_ is the temperature phase transition, and *α* is the exponent related with the degree of diffusion of the phase transition. When the *α* parameter is in the range 1< *α* ≤ 2, the phase transitions assume the diffused form [46]. Figure 6 presents the plots of ln(1/*ε* – 1/*ε*_m_) versus ln (*T* – *T*_m_) for the PZT-type samples in temperature range *T* > *T*_m_ (made for the paraelectric phase). The P-Dy sample shows the highest degree of diffuse phase transition from the series of the analyzed compositions. The *α* parameter for the ceramic samples was calculated according to Equation (1) and presented in Figure 6 and Table 1. 

Compared with undoped PZT materials [20,47], the selected set of admixture affects lowering the temperature of the phase transition, which is attributed to the introduction of the dopants in the PZT crystal lattice. When these doped ions enter the A or B sites of the crystal lattices, lead vacancies and oxygen vacancies are created, resulting in a change in electric conductivity and lowering *T*_m_ [48]. 

**Figure 7:**
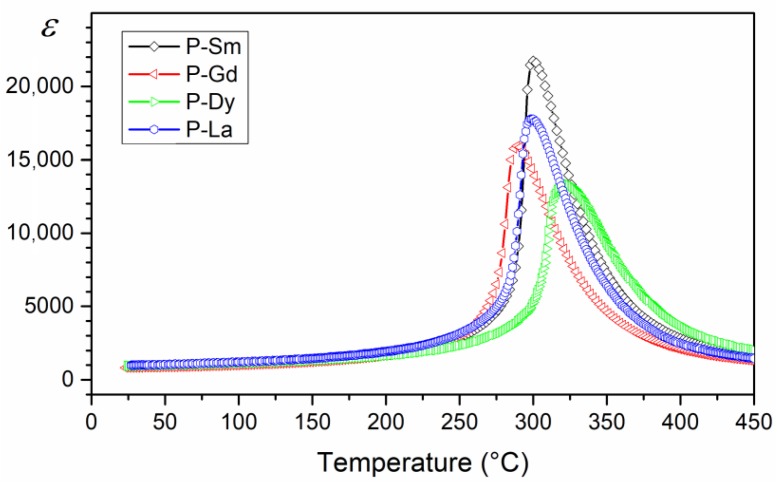
Comparison *ε*(*T*) graph for the four multicomponent PZT-type materials (for 1 kHz).

The summary of temperature graphs *ε*(*T*) for all analyzed compositions (for 1 kHz) is presented in Figure 7. The study shows that the multicomponent ceramic materials of the PZT-type have high electric permittivity values. At room temperature, the compositions obtained have similar electric permittivity values (Table 1). The admixtures of samarium (Sm) and lanthanum (La) introduced into the PZT base composition results in obtaining the material with the highest values of maximum electric permittivity. The PZT-Sm also has the sharpest phase transition. In turn, doping the basic composition of PZT with gadolinium and dysprosium reduces the maximum value of electric permittivity at *T*_m_ and results in a greater degree of diffuse phase transition. The addition of dysprosium, additionally, shifts the *T*_m_ temperature towards higher temperatures.

**Figure 8:**
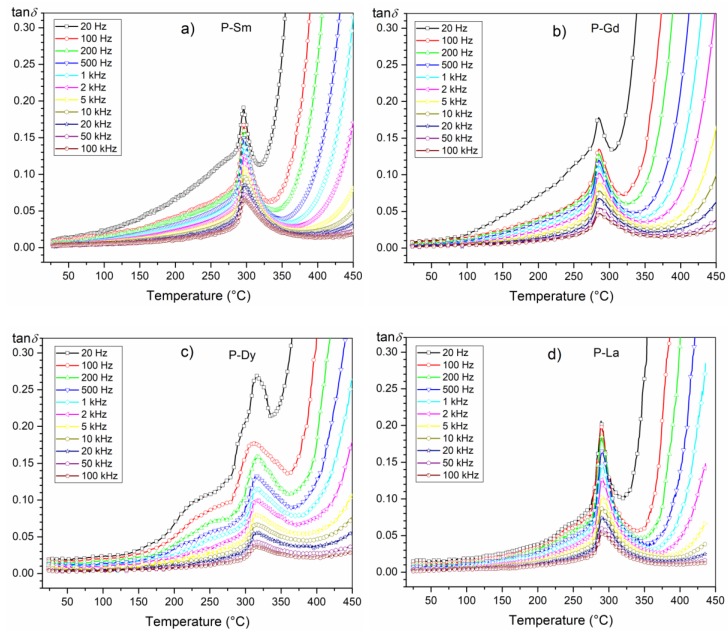
Temperature dependencies of the dielectric loss of the following multicomponent PZT-type samples: (**a**) P-Sm, (**b**) P-Gd, (**c**) P-Dy, (**d**) P-La.

In the case of ferroelectric ceramic materials, the dielectric loss originates from different factors as follows: space charge migration (interfacial polarization contribution), direct current (DC) conduction, and the movement of the molecular dipoles [49]. Multicomponent compositions of the PZT-type have low dielectric loss values in the entire temperature range (Figure 8, Table 1). In the case of compositions with sharp phase transition, i.e., P-Sm and P-La, the tan*δ*(*T*) curves are similar, whereas the dielectric loss values are practically the same. On the graph of tan*δ*(*T*), all of the analyzed compositions show a characteristic peak (i.e., the local maximum of the dielectric loss values occurring just before the phase transition). 

In the perovskite materials at lower frequencies, there are higher values of electric permittivity (Figure 5). The higher values of the electric permittivity at lower frequencies are associated with the presence of various types of polarization, i.e., dipolar, atomic, ionic, and electronic contribution [50].

**Figure 9:**
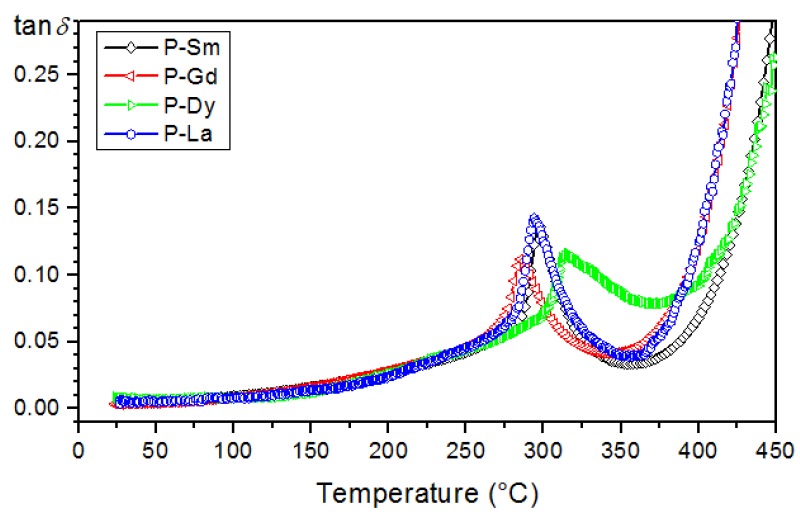
Comparison tan*δ*(T) graph for the four multicomponent PZT-type materials (for 1 kHz).

The summary of tan*δ*(T) graphs for all analyzed compositions (for 1 kHz) is presented in Figure 9. At room temperature, the obtained compositions have similar dielectric loss values (Table 1). At higher temperatures, doping with samarium, gadolinium, dysprosium, or lanthanum does not have a significant effect on the dielectric loss values, and all analyzed compositions show low dielectric loss values throughout the entire measuring area. As the temperature increases, the dielectric loss increases as well. This indicates that the concentration of conduction electrons increases as temperature rises (due to a greater thermal activation of the system). Above 320 °C, a considerable increase of dielectric loss is observed (electric conductivity increase). 

**Figure 10:**
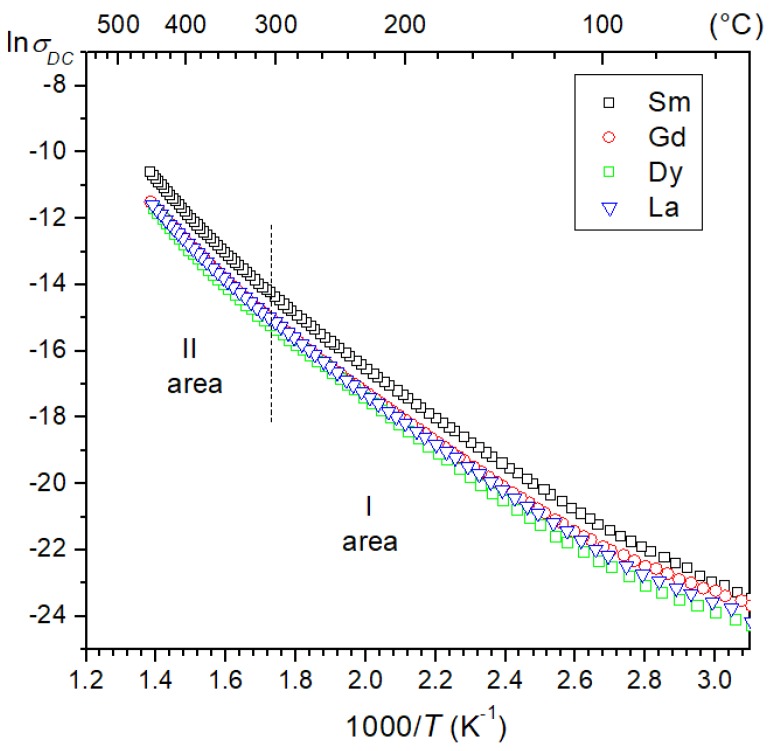
The ln*σ*(1000/*T*) relationship for the four multicomponent PZT-type materials.

Temperature changes in the DC electrical conductivity of the ceramic materials are presented in the form of the ln*σ*_DC_(1000/T) graph (Figure 10). All ceramic compositions show similar waveforms of temperature dependence. As the temperature rises, a slow increase in electrical conductivity in the materials is observed. Changing the *M* admixtures (i.e., Sm, Gd, Dy, and La, in the amount of 0.01) in the base chemical compound does not change the nature of electrical conductivity.

During heating of the perovskite materials, the transformation of one phase into another is most often accompanied by a change in the activation energy of the carriers. It is associated with a change in the bandwidth of forbidden energy or a change in the position of doped levels, as well as other factors such as: change in the polarization value due to the disappearance of spontaneous polarization [51]. These changes are visible on the dependencies ln*σ*_DC_(1000/*T*), in the form of simple temperature sections with varying degrees of slope. The slope changes occurring at a given temperature (depending on the defect of the crystal structure) could be related, for example, to the occurrence of areas with different admixed and intrinsic conductivity in the material. Activation energy was calculated from the slope of ln*σ*_DC_(1000/*T*) plot (two regions of the figure, i.e., lower and higher temperature) and according to the Arrhenius law:

(6) $$\sigma_{DC}=\sigma_{0}e^{-\frac{E_{Act}}{k_{B}T}}$$

where *σ*_0_ is the pre-exponential factor, *k_B_* is the Boltzmann constant, *E_Act_* is the activation energy, and *T* is the absolute temperature [51]. The graphs ln*σ_DC_*(1000/*T*) show changes in the slope of the curves with different values of activation energy below and above phase transition (Figure 10). The calculated activation energy values for all compositions are summarized in Table 1. An increase in activation energy is observed for all tested materials above the phase transition. 

There are many approaches to the mechanism of electrical conductivity in perovskite materials [26,28,43,52,53,54]. Undoped PZT materials have the p-type conductivity (hole type semiconductor) and this occurs because of PbO evaporation from basic chemical composition during the technological process [43]. Due to the space charge, both centers of negative charges and the whole carriers increase violently, and this causes an internal field inside the grains. This field can restrain domain motion (the phenomenon restrain of domain motion reduces the dielectric loss) [43]. According to [53], for undoped PZT material ionized lead vacancies as electron acceptors are formed by $V_{\mathrm{Pb}}\to V_{\mathrm{Pb}}^{\prime }+⊕$, for the first ionization and by $V_{\mathrm{Pb}}^{\prime }\to V_{\mathrm{Pb}}^{\prime \prime }+⊕$, for the second ionization. In the above formulas, $V_{\mathrm{Pb}}$ is a lead vacancy, $V_{\mathrm{Pb}}^{\prime }$ is a singly ionized lead vacancy, $V_{\mathrm{Pb}}^{\prime \prime }$ is a doubly ionized lead vacancy, and ⊕ is the hole. In [52], a different mechanism of electrical conductivity (p-type) in perovskite materials was proposed, in which oxygen vacancies play a major role (analysis of the ratio of empty and filled vacancies $V_{O}^{••}$). Oxygen vacancies can be filled by one electron, two electrons, or they can be empty. If the share of vacancies with two electrons is not dominant, this state corresponds to p-type conductivity [52]. In perovskite materials, the oxygen vacancies are positively charged with respect to the lattice and always appear as single ionized at low temperature (the energy of the first ionization of oxygen vacancies is about 0.1 eV) or as doubly ionized at high temperature (second energy ionization is about 1.4 eV). The thermally liberated electrons contribute to the dielectric relaxation and conduction process [55]. According to [26], PZT is characterized by a disordered Schottky-type structure, where the concentration of lead $V_{\mathrm{Pb}}^{\prime \prime }$ vacancies is much higher than the concentration of oxygen vacancies $V_{O}^{••}$. In the case of doping, ions with a lower degree of oxidation introduced into the A(B) positions of the PZT compound introduce fewer electrons than the ions found in these positions Pb(Zr,Ti) and form holes, that is, they act as acceptors. Acceptor admixtures reduce cross-resistivity (increase of electrical conductivity). 

In the analyzed multicomponent PZT-type ceramic materials there is a mixed mechanism of electrical conductivity associated with the acceptor and donor doping of the main compound. The acceptor dopant is antimony Sb^3+^ and is substituted in the B positions. These substitutions for (Ti,Zr)^4+^ ions create an acceptor level on the B site in the PZT perovskite structure. The ⊕ holes are generated from the acceptors on the way Sb_(Ti,Zr)_^⋅^→Sb_(Ti,Zr)_+ ⊕, where Sb_(Ti,Zr)_ is normal in PZT position and Sb_(Ti,Zr)_ are ionized ions [53]. A different conductivity mechanism occurs as a result of the introduction of donor dopants. The donor dopants are tungsten W^6+^ (substituted in the B position, in place of Ti^4+^/Zr^4+^) and *M*^3+^ (i.e., samarium Sm^3+^, gadolinium Gd^3+^, dysprosium Dy^3+^, or lanthanum La^3+^) substituted in the A position (in place of Pb^2+^). In this case, the donor admixtures contribute by generating excess electrons to the conduction process [53]. Taking into account the whole set of admixtures used, the excess electrons of dopants and their deficiencies partially compensate each other and change the doping effect. The lower activation energy (below 1.0 eV) and a small difference in the *E_Act_* value (in the low-temperature and high-temperature area) for the analyzed compositions could also be the result of the above phenomenon. A low value of activation energy has been observed in many Pb-based ferroelectric complex compounds [31].

**Figure 11:**
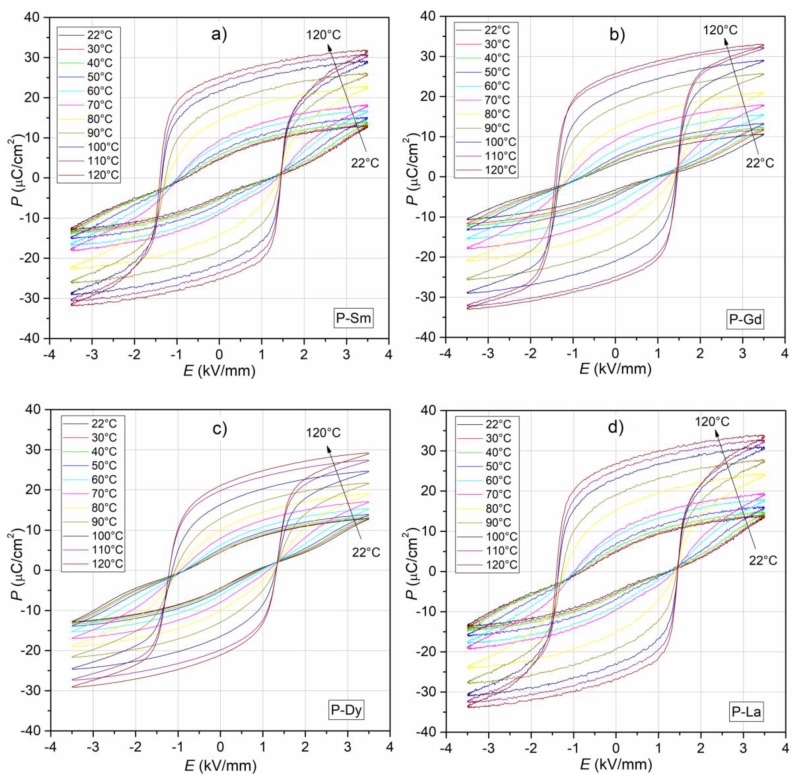
The *P-E* hysteresis loops for the four multicomponent PZT-type materials (in temperature range from 22 to 120 °C, 1 Hz). : (**a**) P-Sm, (**b**) P-Gd, (**c**) P-Dy, (**d**) P-La.

The results of the temperature test of *P-E* hysteresis loops measured for 1 Hz for all composition of the PZT-type materials are depicted in Figure 11. At room temperature, the *P-E* loops are characteristic for dielectric materials with losses [56,57,58]. The hysteresis loops show a weak degree of saturation for the maximum electric field applied to the sample (3.5 kV/mm), and the distinctive narrowing of the loops in their center is characteristic for all samples. At room temperature, the *E_C_* coercive field values assume average values, i.e., 0.94 kV/mm, 0.86 kV/mm, 0.82 kV/mm, and 1.02 kV/mm for the P-Sm, P-Gd, P-Dy, and P-La samples, respectively (Table 1), while the lanthanum-doped sample (P-La) shows the highest residual polarization values (4.92 μC/cm^2^). The tests showed slight differences in the residual polarization values and in the coercive field with the change of the admixture in the base composition (Table 1). 

For all composition, with increasing temperature, the hysteresis loops saturation increases (Figure 11). In the temperature range from 22 to 120 °C, the *P_r_* residual polarization increases significantly (above five-fold), and at 120 °C it is 25.06 μC/cm^2^, 25.67 μC/cm^2^, 21.07 μC/cm^2^, and 26.80 μC/cm^2^ for the P-Sm, P-Gd, P-Dy, and P-La samples, respectively. In the case of the *E_C_* coercive field, in higher temperature, the values change slightly, and at 120 °C the values are 1.43 kV/mm, 1.41 kV/mm, 1.27 kV/mm, and 1.46 kV/mm for the P-Sm, P-Gd, P-Dy, and P-La samples, respectively.

The *P-E* tests showed that the analyzed materials have intermediate properties between ferro-hard and ferro-soft PZT-type ceramic materials (i.e., good dielectric and piezoelectric parameters) [26,27,59]. In the case of the ceramic materials with perovskite structure, the coercive field depends, inter alia, on the grain size of the microstructure. The microstructure of the samples with low values of coercive field is characterized by large grains, whereas the one with high values of coercive field is characterized by small grains. It is attributed to the considerable difficulty of domain polarization reversal in the case of the smaller grains. 

The values of the piezoelectric coefficient, *d*_33_, measured at room temperature are in the range from 263 pC/N to 341 pC/N and are 276 pC/N, 263 pC/N, 268 pC/N, and 341 pC/N for P-Sm, P-Gd, P-Dy, and P-La samples, respectively. The P-La ceramic sample shows the best piezoelectric properties. This type of ceramic material properties is most suitable for microelectronic applications, for example, as an element in actuators or piezoelectric transducers. 

The mixed method of doping of the PZT-type ceramic materials (acceptor and donor doping) has a positive effect on the set of the electrophysical parameters of ceramic materials (Table 1). Donor dopants (i.e., W^6+^, Sm^3+^, Gd^3+^, Dy^3+^, and La^3+^) increase the dielectric and piezoelectric properties, whereas the acceptor dopant (Sb^3+^) increases the stability of the electrophysical parameters. The multicomponent ceramic samples show higher values of piezoelectric parameters as compared with the non-admixed PZT ceramics [48] and the PZT compositions doped with single admixtures, for example, [32,48]. In addition, the suitable selection of the set of admixtures improved the sinterability of the ceramic samples, as well as resulted in obtaining the required material for the poling process.

## 4. Conclusions

In the paper, the results of investigation for four multicomponent ceramic materials with a general chemical formula Pb_0.99_*M*_0.01_((Zr_0.49_Ti_0.51_)_0.95_Mn_0.021_Sb_0.016_W_0.013_)_0.9975_O_3_ (where *M* = Sm^3+^, Gd^3+^, Dy^3+^, and La^6+^) are described. 

The XRD studies have shown that all of the obtained compositions have a structure from a tetragonal system with a P4mm point group, without the presence of other undesirable phases. The microstructure of ceramic samples is characterized by well crystallized grains, with clearly visible grain boundaries. The dielectric tests have shown that the dopants of Dy and Gd introduced into the main compound reduce the values of electric permittivity (the dopant of Dy also significantly blurs the ferroelectric-paraelectric phase transition), while the admixture of La and Sm improve dielectric properties. The *P-E* tests showed that the obtained materials have intermediate properties between ferro-hard and ferro-soft ceramic materials. The best set of dielectric and piezoelectric parameters is exhibited by the composition of P-La (with an admixture of lanthanum).

The mixed doping method (acceptor and donor doping) used in this work has a positive effect on the set of electrophysical properties of ceramic materials. Donor dopants, i.e., W^6+^ (at positions B) and *M*^3+^ = Sm^3+^, Gd^3+^, Dy^3+^, and La^3+^ (at positions A) increase the dielectric and piezoelectric properties, while the acceptor dopant Sb^3+^ (at positions B) increases the time and temperature stability of the electrophysical parameters. The set of admixtures used in this paper improved the sinterability of the samples during the technological process. Ceramic materials with optimal properties and proper microstructure do not create difficulties during the process of polarization (it is possible to apply stronger electric fields to the sample), and thus obtain high values of piezoelectric parameters. 

This research confirms that all of the obtained ceramic compositions show good dielectric and piezoelectric parameters that allow their use in modern micromechatronic and microelectronic applications, for example, as actuators, piezoelectric transducers, and precision microswitches. 

## Figures and Tables

**Table 1:** Electrophysical properties of the multicomponent PZT-type ceramics.

	P-Sm	P-Gd	P-Dy	P-La
*a*_0_ (Å)	4.0313(1)	4.0305(1)	4.0363(1)	4.0314(1)
*c*_0_ (Å)	4.1226(1)	4.1225(1)	4.1249(1)	4.1154(1)
*c*_0_/*a*_0_	1.0226	1.0228	1.0219	1.0208
*δ*_T_	0.015042	0.015159	0.014580	0.013843
*V* (Å)^3^	66.997	66.970	67.202	66.884
*D* (nm)	44	41	27	29
*δ*	5.07	5.88	13.58	12.06
*ξ*	0.17	0.18	0.27	0.26
$\bar{r}$ (μm)	9.2	8.9	8.6	7.6
*T_C_* (°C)	300	290	321	299
*α*	1.73	1.75	1.83	1.74
*ε* at RT	900	820	940	960
tan*δ* at RT	0.0043	0.0033	0.0082	0.0064
*ε*_m_	21,660	15,950	12,290	17,760
tan*δ* at *T*_m_	0.1296	0.1028	0.1106	0.1311
*ρ_DC_* at *T_r_* (Ωm)	1.41 × 10^9^	8.20 × 10^9^	2.55 × 10^9^	1.70 × 10^10^
*E_Act_* in I (eV)	0.61	0.60	0.63	0.62
*E_Act_* in II (eV)	0.93	0.89	0.93	0.89
*P_r_* (μC/cm^2^)	4.69	3.14	4.45	4.92
*P_S_* (μC/cm^2^)	8.58	6.74	8.90	9.71
*E_c_* (kV/mm)	0.94	0.86	0.82	1.02
*k_p_*	0.50	0.48	0.50	0.52
*d*_31_ (pC/N)	157	95	114	119
*g*_31_ × 10^−3^ (Vm/N)	9.95	11.31	11.22	11.59
*Q*_m_	12	26	94	88
*d*_33_ (pC/N)	276	263	268	341

Crystallographic properties: *a*_0_ and *c*_0_, unit cell parameters; *V*, unit cell volume; *δ*_T_, spontaneous deformation of the tetragonal elementary cell; *D*, minimal possible size of particles; *δ*, dislocation density; *ξ*, microstrain. Dielectric parameters: *T*_m_, the temperature phase transition; *α*, exponent related with the degree of diffusion of the phase transition; *ε*, electric permittivity; tan*δ*, dielectric loss; *ε*_m_, maximum electric permittivity at the *T*_m_ temperature. Electric conductivity: *ρ_DC_*, DC resistance; *E_Act_*, activation energy. Ferroelectric parameters: *P_r_*, residual polarization; *P_S_*, spontaneous polarization; *E_c_*, coercive field. Piezoelectric parameters: *k_p_*, electromechanical coupling factor; *d*_31_ and *g*_31_, piezoelectric coefficients; *Q*_m_, mechanical quality facto; *d*_33_, piezoelectric coefficient.

**Table 2:** Theoretical and experimental percentages of elements of PZT-type ceramics.

	P-Sm		P-Gd		P-Dy		P-La	
	Th. (%)	Ex. (%)	Th. (%)	Ex. (%)	Th. (%)	Ex. (%)	Th. (%)	Ex. (%)
PbO	67.79	69.87	67.84	69.46	67.80	70.36	67.89	69.91
*M*_2_O_3_	0.54	0.87	0.55	0.69	0.56	0.83	0.50	0.50
ZrO_2_	17.60	15.47	17.57	15.55	17.60	14.98	17.58	16.05
TiO_2_	11.87	11.48	11.85	12.05	11.87	11.54	11.86	11.71
MnO_2_	0.56	0.53	0.56	0.55	0.55	0.50	0.56	0.45
Sb_2_O_3_	0.71	0.52	0.71	0.47	0.71	0.51	0.71	0.64
WO_3_	0.93	1.26	0.92	1.23	0.91	1.28	0.92	0.74
